# Effectiveness of CT perfusion in posterior circulation stroke: evaluation of perfusion abnormalities and associated clinical signs

**DOI:** 10.1007/s00415-025-12933-4

**Published:** 2025-02-22

**Authors:** Giovanni Furlanis, Edoardo Ricci, Miloš Ajčević, Filippo Spigariol, Emanuele Vincis, Gabriele Prandin, Laura Mancinelli, Federica Palacino, Magda Quagliotto, Paola Caruso, Maja Ukmar, Marcello Naccarato, Paolo Manganotti

**Affiliations:** 1 Clinical Unit of Neurology, Department of Medicine, Surgery and Health Sciences, University Hospital and Health Services of Trieste - ASUGI, University of Trieste, Strada di Fiume, 447, 34149 Trieste, Italy; 2 Department of Engineering and Architecture, University of Trieste, Via A. Valerio, 10, 34127 Trieste, Italy; 3 Radiology Unit, Department of Medicine, Surgery and Health Sciences, University Hospital and Health Services of Trieste - ASUGI, University of Trieste, Strada di Fiume, 447, 34149 Trieste, Italy

**Keywords:** Posterior circulation stroke, CT perfusion, Neuroimaging, Multimodal CT imaging, Stroke

## Abstract

**Purpose:**

Acute posterior circulation stroke (PCS) is characterized by often non-specific clinical signs, with neuroimaging playing a pivotal role in assessment in the emergency setting. The aim of this study was to investigate the effectiveness of CT Perfusion (CTP) maps in detecting acute PCS and to identify clinical factors associated with perfusion abnormalities.

**Methods:**

We retrospectively analyzed clinical and radiological data of consecutive patients with acute PCS admitted to our Stroke Unit that underwent CTP. Follow-up NECT or MRI was performed to confirm the diagnosis of PCS. The effectiveness of CTP to identify PCS was evaluated as the ratio of the CTP in which perfusion abnormalities, compatible with an ischemic event, were present in at least one CTP map among MTT, CBF, TTP, and CBV. Multivariate logistic regression analysis was conducted to identify clinical factors associated with perfusion abnormalities.

**Results:**

CTP showed alterations in 69 of 107 PCS (64.5%) included in final analysis and MTT proved to be the most sensitive. Multivariate analysis showed that atrial fibrillation (OR = 8.571, CI 95% 2.224–33.037, *p* = 0.002), dyslipidemia (OR = 0.285, CI 95% 0.100–0.814, *p* = 0.019), visual field deficits (OR = 3.372, CI 95% 1.020–11.150, *p* = 0.046), and higher neurological deficit (NIHSS > 5) (OR = 4.054, CI 95% 1.147–14.331, *p* = 0.030) were significantly associated with perfusion abnormalities on CTP.

**Conclusion:**

CT perfusion can be a valuable resource for detecting acute PCS showing a moderately high positivity rate, higher than that of NECT alone or CTA. These findings contribute to the growing body of evidence supporting the use of perfusion imaging in acute posterior circulation stroke.

## Introduction

Posterior circulation stroke (PCS) is defined as the ischemic stroke of the brain’s portions vascularized by the posterior circulation. With an incidence of 18 cases per 100,000 person/year, PCS makes up for 20–25% of all ischemic strokes [1]. It has a mortality at 1 month of 3.6–11% and a disability at 3 months of 6.9–19.8% [2, 3]. The most common symptoms and signs of PCS are unilateral force deficit, facial paralysis, ataxy, dysarthria, nystagmus, vertigo, nausea and vomit, headache, and altered consciousness [3–5]. Several syndromes have been identified for different affected ischemic regions [1–6]. The stroke severity and diagnosis are traditionally based on the National Institute of Health Stroke Scale (NIHSS). However, PCS is intrinsically difficult to diagnose, due to the various sets of signs and symptoms, the presence of non-focal symptoms, and the low prevalence of specific signs [3]. For these reasons, other clinical scales of evaluation were created aiming at a better characterization of PCS compared to the traditional NIHSS. These scales are the expanded NIHSS (e-NIHSS) [7] and the Israeli Vertebro-Basilar Stroke Scale (IVBSS) [8].

As for any stroke, the size of the damage caused by the PCS is determined by the size of the occluded vessel, the presence of a collateral circulation in that region, the presence of an ischemic penumbra, and the time elapsing between the ischemic event and a possible recanalization therapy. Neuroimaging plays a pivotal role in the diagnosis and the decision-making. In the past years, the new imaging techniques of magnetic resonance imaging (MRI) and computed tomography perfusion (CTP) have become crucial in identifying patients who can benefit from a prompt reperfusion treatment. Once the area of ischemic penumbra is identified, the reduced-perfusion area may be saved by quickly restoring its vascularization, while also predicting the patient’s functional outcome [9–11].

The dogma of ischemic stroke therapy is “time is brain.” The main reperfusion choices for acute ischemic stroke, intravenous thrombolytics and endovascular thrombectomy are time-dependent techniques: the former is applicable in the first 4.5 h and the latter after 6 h after stroke. Yet in recent years, the mantra “penumbra is brain” is gaining importance in treatment decisions, since the presence of “salvageable hypoperfused” tissue can make a difference in patient management regardless of symptom onset [11, 12]. The new approach is applicable only by utilizing perfusion imaging techniques. Although MRI has a higher contrast resolution in identifying ischemic stroke, this technique has some limitations in terms of availability and affordability, which is why CTP is increasingly gaining ground as a commonly adopted alternative in the acute phase. It has been widely demonstrated that CTP plays a key role in the selection of reperfusion therapy in wake-up stroke patients, stroke with unknown onset or out of conventional therapeutic windows [13, 14]. Neuroimaging studies indicate that CTP may exhibit low and variable sensitivity in detecting areas of altered blood flow in the posterior territories and that its specificity is lower than non-enhanced CT (NECT) [15].

Therefore, the aim of this study was to investigate the effectiveness of CTP maps in detecting PCS in the acute setting, as well as the clinical factors associated with the presence of perfusional alterations.

## Methods

### Study population

We retrospectively analyzed clinical and radiological data of patients with a diagnosis of posterior ischemic stroke admitted to the Stroke Unit of the University Hospital of Trieste (Italy) between April 2016 and March 2022 who underwent evaluation with NECT, CT angiography (CTA), CTP, and follow-up NECT. The diagnosis of ischemic stroke in the posterior circulation was confirmed through a follow-up NECT showing an infarct lesion. In cases where no lesion was visible on NECT or where the diagnosis was unclear, an MRI was performed to confirm the presence of a posterior circulation stroke. We included patients who were 18 years of age or older and both genders. Patients with hemorrhagic stroke, stroke mimics, ischemic strokes of the anterior circulation, and those who did undergo a multi-parametric CT assessment (NECT, CTA, and CTP) were excluded. Our neuroimaging protocol included NECT, CTA, CTP, and control NECT at 24/48 h for all patients with NIHSS < 6 and symptom onset < 4.5 h, with NIHSS > 6 and symptom onset < 6 h and in all wake-up strokes. The following data of included patients were collected: (1) demographic details (age, sex); (2) etiology based on TOAST [16] classification (atherothrombotic, cardio-embolic, lacunar infarction, cryptogenic, other causes); (3) risk factors (smoke, hypertension, diabetes, dyslipidemia, atrial fibrillation, ischemic cardiopathy); (4) clinical features (wake-up stroke, National Institutes of Health Stroke Scale (NIHSS), patient with NIHSS > 5 (the individual items of the NIHSS were assessed and we dichotomized this scale: NIHSS > 5 for moderate and severe strokes and NIHSS < 5 for minor strokes), expanded NIHSS (e-NIHSS) [7], and Israeli Vertebro-Basilar Stroke Scale (IVBSS) [8] scores at admission); (5) baseline imaging (positive baseline NECT, pc-ASPECT [17], positive CTP, CBV pc-ASPECT, and MTT pc-ASPECT, lesion volume at CTP, positive CTA and localization of occlusion); (6) prognostic evaluation with mRS at discharge and at 3 months; and (7) imaging outcome (positive NECT control, pc-ASPECT, and lesion volume at NECT).

### Neuroimaging acquisition and analysis

CT standardized protocol at admission involved non-enhanced CT, single-phase CT angiography, and CTP. Follow-up imaging was performed with NECT at 22–36 h after admission. All CT imaging was performed with a 256-slices CT scanner (Brilliance iCT; Philips Medical Systems, Best, Netherlands). NECT was acquired with 120 kV, 400–450 mAs, at a slice thickness of 0.9 and reconstructed at 5 mm. CTP acquisition protocol involved injections of intravenous contrast medium administered at an injection rate of 4 ml/s and a total scanning time of 60 s. The exposure parameters used were 80 kVp and 150–200 mA s, and a three-dimensional axial acquisition on the whole-brain volume with a reconstruction of the slices set to 5 mm was performed. CTP data processing was performed using Extended Brilliance Workstation v 4.5 (Philips Medical Systems, Best, Netherlands) and a home-made code developed in Mathlab (MathWorks Inc., Natick, MA) [17–19]. Deconvolution-based methods were used to calculate perfusion maps, namely mean transit time (MTT), cerebral blood volume (CBV), and cerebral blood flow (CBF). Models of the time/attenuation curves were obtained by curve fitting using least mean squares method, and the MTT map was subsequently calculated via a closed-form deconvolution operation using the time/concentration curve of a particular voxel and the arterial input function. For each voxel, the CBV map was calculated from the area under the time/concentration curves. CBF map was consequently calculated as a ratio between CBV and MTT. Time to peak (TTP) was also extracted from the time-attenuation curve. Ischemic volumes were calculated as described in the previous study [11]. The presence of perfusion abnormalities, compatible with an ischemic event, was qualitatively assessed by an experienced neuroradiologist and experienced neurologists from the Stroke Unit (MU, MN and GF). Subsequently, we divided the patients into two groups based on the presence or absence of identified alterations in the perfusion maps. The CTP + group included subjects with at least one CTP map where an alteration was identified, while the CTP − group consisted of cases in which no alterations were identified through visual inspection.

### Statistical analysis

We performed all statistical analyses using SPSS Statistics 23 (IBM, Armonk/NY, USA). Kolmogorov–Smirnov test was used to evaluate the normal distribution of variables. Continuous variables with a normal distribution are presented as mean and standard deviations (SDs), those with a skewed distribution as median and interquartile ranges (IQRs) indicating the first and third quartiles, and categorical variables as counts and percentages (%). The two subgroups were defined according to the presence or absence of alterations to the CTP perfusion maps, as previously described. Differences between groups were tested with Student’s *t* test for normally distributed continuous variables, Mann–Whitney *U* test for skewed variables, and Pearson’s Chi-square for categorical variables. The univariate analysis (binary logistic regression) was performed to investigate clinical factors associated with the presence of perfusion alterations on CTP. Specifically, the following factors were considered: age, sex, smoking, hypertension, diabetes, dyslipidemia, atrial fibrillation, ischemic cardiopathy, WUS, NIHSS > 5, altered consciousness, best gaze deficit, visual field deficit (VFD), dysarthria, motor deficit, limb ataxia, sensory alteration, and positive NECT at baseline. Subsequently, a multivariate logistic regression analysis was conducted using variables with *p* values < 0.05 from the univariate analysis. The results are presented as odds ratios (OR), 95% confidence intervals (95% CI), and *p* values. A *p* value < 0.05 was considered statistically significant.

## Results

### Study population

**Fig. 1:**
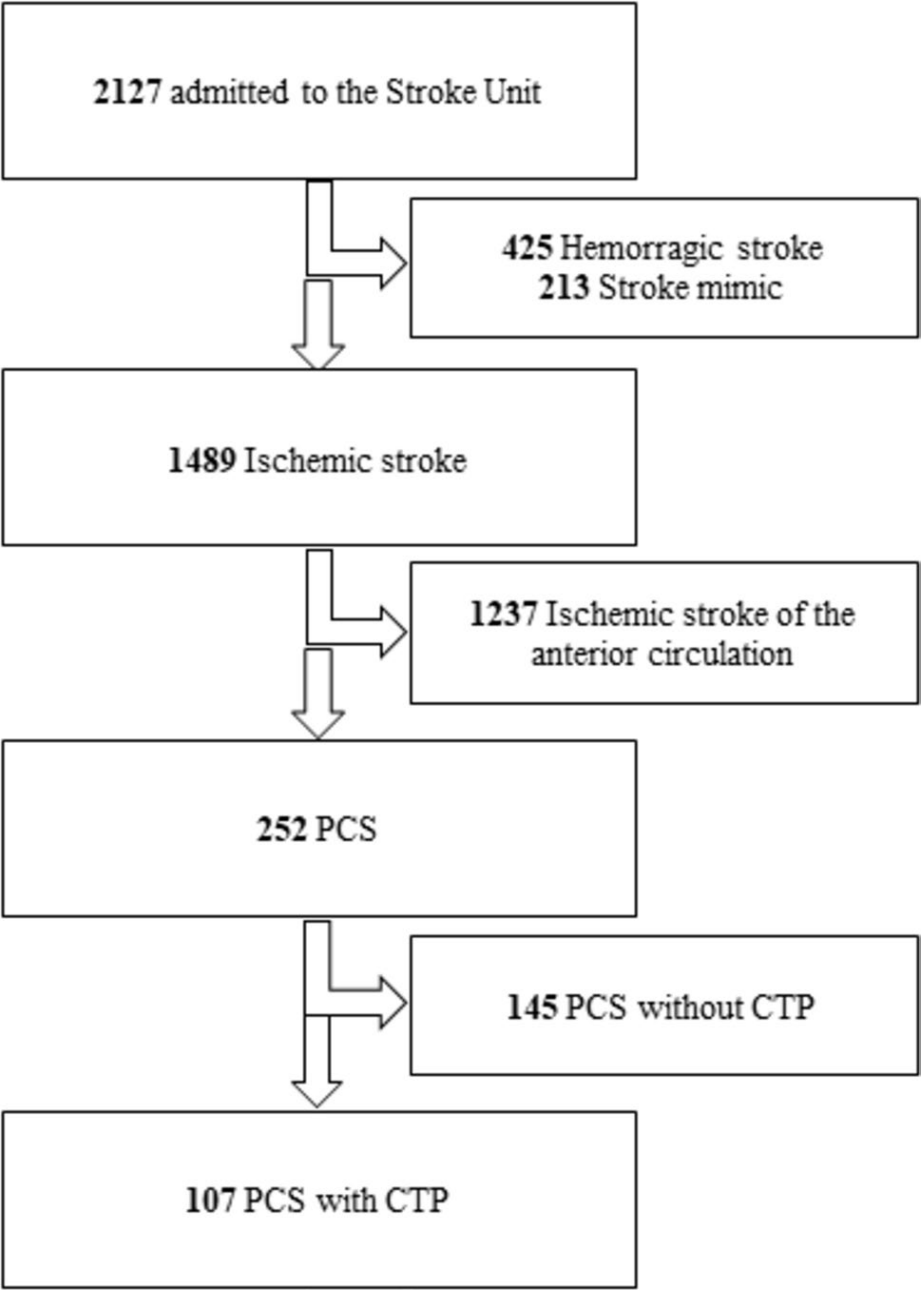
Study flow diagram

During the study period from April 2016 to March 2022, a total of 2127 patients were admitted to our Stroke Unit. Among these, 1489 were diagnosed with ischemic stroke, as confirmed by clinical assessment and follow-up neuroimaging (NECT or MRI). Of these, 252 were posterior circulation strokes (PCS). The 107 PCS patients who arrived within the time window for reperfusion treatment underwent multimodal CT assessment (NECT, CTA, and CTP) and were included in the study (Fig. 1).

Sixty-nine patients (64.5%) had an alteration in the CTP perfusion maps. The mean age of our patient cohort was 69 ± 15.3 years, with females accounting for 42.1%. Clinical and demographic data, TOAST classification, and risk factors of our groups are reported in Table 1. Twenty-five percent of the patients had a positive NECT at baseline, with a median pc-ASPECTS of 10, with a median CBV pc-ASPECT of 10 and MTT pc-ASPECT of 9. CTP analysis suggested a total volume of hypoperfused tissue of 44.3 mL, a penumbra volume of 23 mL, and core of 13.4 mL. An occlusion at CTA was documented in 60 patients (56.1%), in particular 20 patients had an occlusion of the vertebral artery, 22 of the basilar artery, 24 of the posterior cerebral artery (the remaining data on the location of the occlusion can be seen in Table 1). The average time from stroke onset to neuroimaging assessment in our cohort was 190 min.

**Table 1:** Participants’ demographics and characteristics

	n = 107
Demography	
Age (years)	69 ± 15.3
Females [n (%)]	45 (42.1)
TOAST classification [n (%)]	
Cardio-embolic	37 (34.6)
Atherothrombotic	24 (22.4)
Lacunar	5 (4.7)
Cryptogenic	31 (29)
Other cause	10 (9.3)
Risk factors [n (%)]	
Smoke	31 (29)
Hypertension	81 (75.7)
Diabetes	21 (19.6)
Dyslipidemia	56 (52.3)
Atrial fibrillation	37 (34.6)
Ischemic cardiopathy	19 (17.8)
Clinical features	
WUS [n (%)]	18 (16.8)
NIHSS baseline	5 (2−10)
NIHSS > 5	49 (45.8)
e-NIHSS baseline	5 (3−11)
IVBSS baseline	10 (6−20)
Imaging baseline	
Positive NECT baseline [n (%)]	26 (24.3)
pc-ASPECT	10 (10−10)
Positive CTP [n (%)]	69 (64.5)
pc-ASPECT CBV	10 (9−10)
pc-ASPECT MTT	9 (8−10)
Lesion volume CTP (cm^3^)	
Total	45.167 (22.21−67.24)
Core	13.86 (4.43−25.42)
Penumbra	34.8 (12.82−53.45)
Positive CTA [n (%)]	
Location of occlusion [n (%)]	60 (56.1)
VA	20 (33.3)
BA	22 (36.7)
PCA	24 (40)
PICA	7 (11.7)
AICA	1 (1.7)
Clinical outcomes	
NIHSS discharge	1 (1 − 3)
mRS discharge	2 (1 − 4)
mRS at 3 months	2 (1 − 4)
Good outcome (mRS ≤ 2) [n (%)]	61 (57)
Mortality [n (%)]	13 (12.1)
Imaging outcome	
Positive NECT control [n (%)]	65 (60.7)
pc-ASPECT	9 (8 − 10)
Lesion volume	9.9 (2–28.45)

Participants reported age (y), sex (females, %), TOAST classification (%), history of hypertension (HTN, %), diabetes (DM, %), dyslipidemia (%), smoking (%), obesity (%), atrial fibrillation (AF, %), ischemic cardiomyopathy (ICM, %), wake-up stroke (%) NIHSS at baseline, e-NIHSS at baseline, IVBSS at baseline, positive NECT and pc-ASPECTS in NECT at baseline, in CTP at baseline, volumes of the lesion in CTP, positive CTA and location of the lesion (%), site of occlusion at CTA (%), clinical outcomes with NIHSS, mRS discharge and mRS at 3 months, positive NECT in the control (%) and lesion volume

### Comparison of demographic, clinical, and radiological characteristics of CTP + and CTP − patients

CTP + patients were older than CTP − ones (77 years vs 65 years; *p* = 0.008), while sex was not statistically different. Concerning the etiology, based on the TOAST classification, the cardio-embolic cause was more represented in CTP + strokes (47.8% vs 10.5%; *p* < 0.001). Lacunar strokes were more represented in the group CTP − (13.1% vs 0%; *p* = 0.005). Atrial fibrillation was significantly more represented in the group CTP + (44.1% vs 10.5%; *p* < 0.001), while dyslipidemia was more represented in the group CTP − (71.1% vs 42.6%; *p* = 0.005). Eighteen PCS patients had a wake-up stroke and thirteen of them had alterations in the CTP perfusion maps (72.2% PCS-WUS CTP + vs 62.9% of PCS-non-WUS CTP + ; *p* = 0.452). A greater NIHSS (6 vs 3; *p* = 0.001), e-NIHSS (7 vs 4; *p* = 0.002), IVBSS (12 vs 6; *p* = 0.001), as well as patients with a major neurological deficit (NIHSS > 5) (39 vs 10; *p* = 0.003) are more represented in the CTP + group. In addition, visual field deficits (52.2% vs 15.8%; *p* < 0.001) and motor plane alterations (66.6% vs 42.1%; *p* = 0.014) are more represented in the CTP + group.

We also compared different radiological features between the two groups to highlight the presence of differences, taking into account the results of admission NECT, pc-ASPECT and CTA. The presence of signs of ischemia on the NECT at admission was higher in the CTP + group (22 vs. 4, *p* = 0.018). Furthermore, a higher result was found between positive CTA (72.4% vs 26.3%; *p* < 0.001) and positive CTP, particularly for posterior cerebral artery occlusion (37.6% vs 0%; *p* < 0.001) and basilar artery occlusion (28.9% vs 5.2%; *p* = 0.004). In our cohort, 44 patients exhibited cerebellar involvement, and of these, 32 (72.7%) demonstrated perfusion changes on CTP. Higher scores in NIHSS at discharge (2 vs 0; *p* < 0.001), mRS at discharge (4 vs 1; *p* < 0.001) and mRS after 3 months (3 vs 1; *p* = 0.001) are evident in the CTP + group. An infarct lesion documented on control NECT was more frequent in the CTP + group (79.7% vs 47.3%; *p* = 0.001). All these data are presented in Table 2. Table 3 reports the rates of individual alterations (CTP +) observed in CTP maps (CBV, CBF, MTT, and TTP). The MTT map was the most sensitive for detecting perfusion changes consistent with posterior circulation ischemic lesions.

**Table 2:** Comparison of demographics and clinical characteristics of patients with alteration of CTP and without alterations. Data are presented as means ± sd, medians (IQR) and frequencies

	CTP + n = 69	CTP − n = 38	p value
Demography			
Age (years)	77 (66–82)	65 (51–76)	**0.008**
Females [n (%)]	32 (46.4)	13 (34.2)	0.222
TOAST classification [n (%)]			
Cardio-embolic	33 (47.8)	4 (10.5)	** < 0.001**
Atherothrombotic	15 (21.7)	9 (23.6)	0.817
Lacunar	0 (0)	5 (13.1)	**0.005**
Cryptogenic	16 (23.2)	15 (39.5)	0.076
Rare causes	4 (5.8)	6 (15.7)	0.161
Risk factors [n (%)]			
Smoke	20 (29.4)	11 (28.9)	0.960
Hypertension	54 (77.9)	28 (73.6)	0.621
Diabetes	13 (19.1)	8 (21)	0.811
Dyslipidemia	29 (42.6)	27 (71.1)	**0.005**
Atrial fibrillation	33 (47.8)	4 (10.5)	** < 0.001**
Ischemic cardiopathy	14 (20.5)	5 (13.1)	0.339
Clinical features			
WUS [n (%)]	13 (19.1)	5 (13.2)	0.433
mRS at admission	0 (0–0)	0 (0–0)	0.642
NIHSS baseline	6 (3–12)	3 (2–6)	**0.001**
NIHSS > 5	39 (56.5)	10 (26.3)	**0.003**
e-NIHSS baseline	7 (4–14)	4 (3–6)	**0.002**
IVBSS baseline	12 (6–22)	6 (4–10)	**0.001**
Alteration of level of consciousness	12 (17.4)	2 (5.3)	0.131
Best gaze deficit	40 (57.9)	19 (50.6)	0.428
Visual fields deficit (VFD)	36 (52.2)	6 (15.8)	** < 0.001**
Dysarthria	44 (63.7)	20 (52.6)	0.261
Motor deficit	46 (66.6)	16 (42.1)	**0.014**
Limb ataxia	29 (42)	15 (39.5)	0.797
Sensory alteration	21 (30.4)	9 (23.7)	0.457
Imaging			
Positive NECT baseline [n (%)]	22 (32.8)	4 (10.5)	**0.018**
pc-ASPECT	10 (9–10)	10 (10–10)	**0.016**
Positive CTP [n (%)]	69 100)	0 (0)	**/**
pc-ASPECT CBV	9 (8–10)	10 (10–10)	** < 0.001**
pc-ASPECT MTT	9 (8–9)	10 (10–10)	** < 0.001**
Lesion volume CTP (cm^3^)			
Total	45.167 (21.74–67.32)	0 (0)	/
Core	13.86 (4.11–25.74)	0 (0)	**/**
Penumbra	34.8 (12.64–54.41)	0 (0)	**/**
Positive CTA [n (%)]	50 (72.4)	10 (26.3)	** < 0.001**
Location of occlusion [n (%)]			
VA	15 (21.7)	7 (18.4)	0.804
BA	20 (28.9)	2 (5.2)	**0.004**
PCA	26 (37.6)	0 (0)	** < 0.001**
PICA	5 (7.2)	2 (5.2)	0.694
AICA	0 (0)	1 (2.6)	0.355
SCA	7 (10.1)	0 (0)	**0.042**
Treatment			
Thrombolysis [n (%)]	42 (60.8)	26 (68.4)	0.437
Symptoms recognition-treatment (min)	180 (140–228)	205 (118–241)	0.448
Thrombectomy [n (%)]	16 (23.2)	3 (7.9)	**0.048**
Symptoms recognition-treatment (min)	335 (225–345)	370 (260–657)	0.727
Partial recanalization [n (%)]	4 (25)	1 (33.3)	
Complete recanalization [n (%)]	6 (37.5)	2 (66.6)	
Clinical outcomes			
NIHSS discharge	2 (0–4)	0 (0–1)	** < 0.001**
mRS discharge	4 (1–5)	1 (1–3)	** < 0.001**
mRS at 3 months	3 (1–5)	1 (1–2)	**0.001**
Mortality [n (%)]	11 (15.9)	2 (5.2)	0.131
mRS favorable	31 (45)	30 (79)	**0.001**
Imaging outcome			
Positive NECT control [n (%)]	55 (79.7)	18 (47.3)	**0.001**
Lesion volume	15.75 (4.69–29.02)	0.804 (0.415–5.016)	**0.009**

Bold values for* p* < 0.05

Participants reported age (y), sex (females, %), TOAST classification (%), history of hypertension (HTN, %), diabetes (DM, %), dyslipidemia (%), smoking (%), obesity (%), atrial fibrillation (AF, %), ischemic cardiomyopathy (ICM, %), wake-up stroke (%) NIHSS at baseline, e-NIHSS at baseline, IVBSS at baseline, positive NECT and pc-ASPECTS in NECT at baseline, in CTP at baseline, volumes of the lesion in CTP, positive CTA and location of the lesion (%), site of occlusion at CTA (%), clinical outcomes with NIHSS, mRS discharge and mRS at 3 months, positive NECT in the control (%) and lesion volume

**Table 3:** Characteristics of the CTP abnormalities

	n = 107
CBV ischemic alteration	53 (49.5)
MTT ischemic alteration	69 (64.5)
TTP ischemic alteration	66 (61.7)
CBF ischemic alteration	63 (58.8)

Cerebral blood volume (CBV), mean transit time (MTT), time to peak (TTP) and cerebral blood flow (CBF)

### Clinical predictors of CTP + 

In univariate regression analysis, age (OR = 1.033, CI 95% 1.006–1.062, *p* = 0.018), atrial fibrillation (OR = 7.792, CI 95% 2.495–24.329, p < 0.001), dyslipidemia (OR = 0.303, CI 95% 0.129–0.709, *p* = 0.006), visual field deficit (OR = 5.818, CI 95% 2.158–15.684, *p* = 0.001), motor deficit (OR = 2.750, CI 95% 1.217–6.217, *p* = 0.015), NIHSS > 5 (OR = 3.640, CI 95% 1.533–8.643, *p* = 0.003), and presence of lesion at NECT baseline (OR = 3.979, CI 95% 1.256–12.605, p = 0.019) showed potential association with the CTP + . These variables were included in the multivariate analysis (Table 4) and it was observed that atrial fibrillation (OR = 8.571, CI 95% 2.224–33.037, *p* = 0.002), dyslipidemia (OR = 0.285, CI 95% 0.100–0.814, *p* = 0.019), visual field deficits (OR = 3.372, CI 95% 1.020–11.150, *p* = 0.046), and a higher neurological deficit (NIHSS > 5) (OR = 4.054, CI 95% 1.147–14.331, *p* = 0.030) had a significant association with CTP + .

**Table 4:** Logistic multivariate regression for patients with CTP alterations

	Multivariate analysis	
	OR (95% CI)	*p value*
Age (per 1 y increase)	1.006 (0.974–1.040)	0.710
Atrial fibrillation	8.571 (2.224–33.037)	**0.002**
Dyslipidemia	0.285 (0.100–0.814)	**0.019**
Visual field deficit	3.372 (1.020–11.150)	**0.046**
Motor deficit	0.791 (0.236–2.643)	0.791
NIHSS > 5	4.054 (1.147–14.331)	**0.030**
NECT positivity at baseline	3.296 (0.874–12.435)	0.078

Age (years), atrial fibrillation (AF), dyslipidemia, visual field deficit, motor alteration, patients with NIHSS > 5 and NECT at baseline positivity

*p* values (*p*) for multivariate analysis

Bold values for *p* < 0.05

## Discussion

Acute ischemic stroke of posterior circulation represents 20–25% of all ischemic strokes [1]. The clinical and radiological heterogeneity of this pathological entity has long challenged physicians in identifying and treating it. The main finding of the study is that CTP can detect PCS with a moderately high positivity rate and it can be a useful tool in emergency settings. Among the CTP maps, MTT emerged as the most sensitive for identifying PCS.

In recent years, the literature has often suggested that CTP does not have high sensitivity to detect strokes of the posterior circulation. In particular, one group suggested a sensitivity ranging between 39 and 61% in the posterior cranial fossa, while another group outlined a sensitivity of around 41.4% [20, 21]. In our study, we found CTP hypoperfusion abnormalities in 69/107 patients (64.5%). Our result is probably due to a detailed view of the individual perfusion maps and an extensive application of multimodal CT including CTP in all patients admitted to our Stroke Unit. The analysis of MTT, CBV, CBF and TTP maps is known to lead to an increased sensitivity in detecting ischemic lesions by CTP [21]. Our study also shows that the MTT map is the most sensitive in detecting perfusion abnormalities in patients with PCS.

Thus, CTP may provide valuable complementary information, on perfusion alterations, to those provided by NECT and CTA. This can support the clinician in decision-making when MRI assessment is not available or feasible, especially for identifying patients who are candidates for reperfusion treatment. While CTP is generally less sensitive than MRI due to limitations in imaging of the posterior cranial fossa, it offers a clear advantage due to its shorter execution time, wider availability, and applicability to patients who cannot undergo MRI imaging. This has a clear implication in the clinical practice and management of the patient with suspected PCS in the hyper-acute setting.

**Fig. 2:**
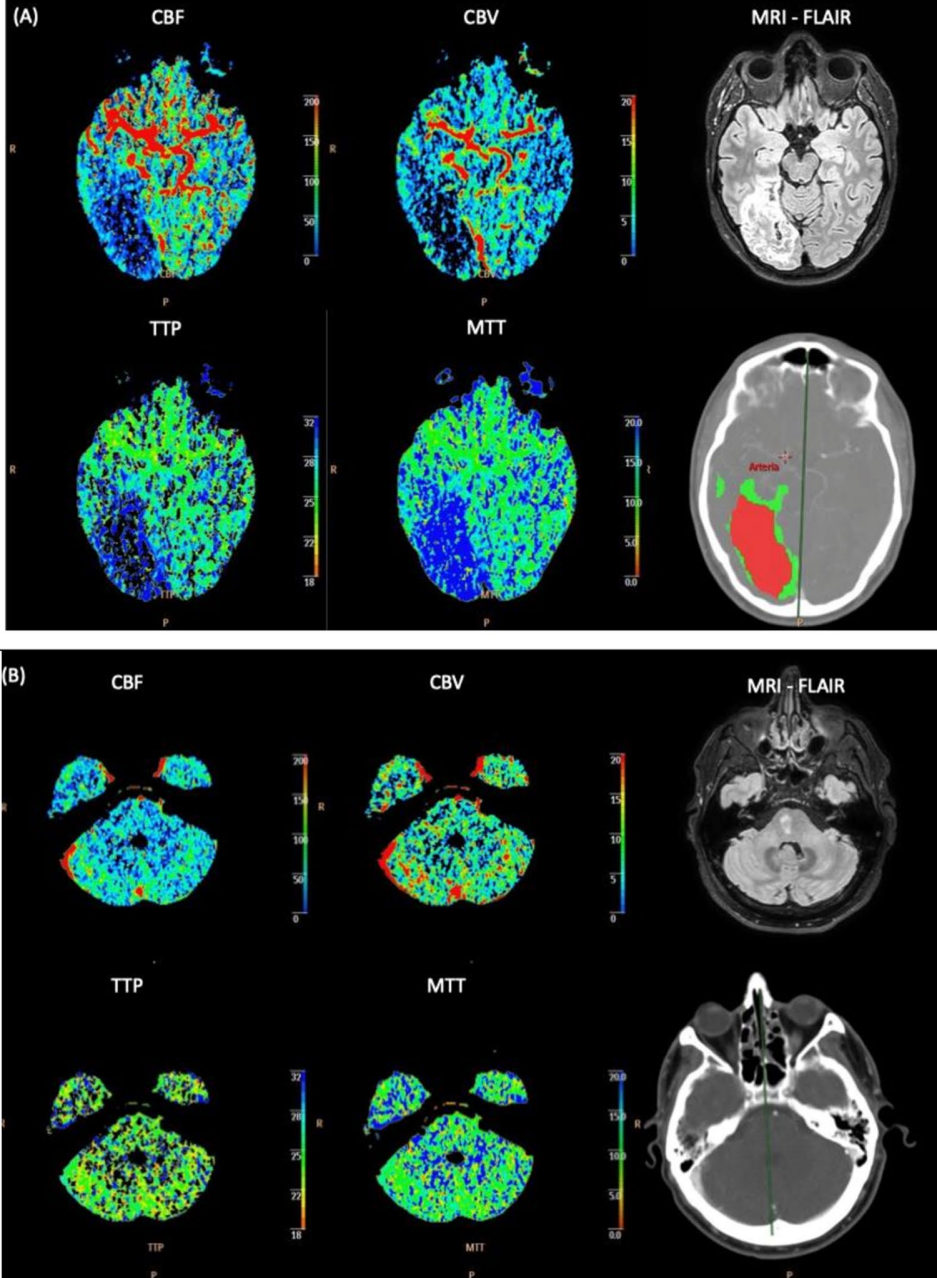
**A** A 43-year-old man with a history of atrial fibrillation presented to the emergency department about an hour after the onset of headache, visual field deficit, vertigo, unsteady gait, and left hemiparesis (NIHSS 10). At NECT, there was evidence of a blurred loss of cortico-subcortical differentiation in the right occipital area (ASPECTS 9); at CTA, there was occlusion of the P2 tract of the right posterior cerebral artery. Finally, evidence of increased rates in MTT and TTP and decreased rates in CBF and CBV was found at CTP in the right occipital area compatible with a large core and minimal penumbra. Control MRI performed 5 days after the acute event confirmed the known ischemic lesion. **B** A 42-year-old man, with a history of dyslipidemia, presented to the emergency department about 3 h after the onset of gait ataxia, hypoesthesia in the face, and left upper limb and mild dysarthria (NIHSS 2). At NECT, CTA, and CTP, there was no evidence of alterations in the acute phase. Control MRI performed 5 days after the acute event documented a 15 mm × 8 mm ischemic vascular lesion in the right paramedian pons

The results of this study showed the association between clinical factors linked to the presence of perfusion alterations on CTP included atrial fibrillation, severe neurological deficit (NIHSS > 5), visual field deficit, and the absence of dyslipidemia. Therefore, in cases where these clinical features are present, in particular severe neurological deficit and visual field deficit but CTP findings are negative, clinicians may take into consideration alternative diagnoses or conduct further diagnostic evaluations. One of the aspects that we prioritize is facilitating clinicians in the diagnosis of posterior circulation strokes through the advanced diagnostic means at our disposal (multi-parametric CT) integrated with semeiotics and clinical signs of the brain areas involved. Examples illustrating two clinical presentations studied with CTP are reported in Fig. 2.

The results of this study showed that AF detection in patients with stroke of the posterior circulation was strongly associated with alterations in perfusion maps at CTP (OR = 8.571, *p* = 0.002). Atrial fibrillation (AF) and stroke share an intricate causal link in patients with worse clinical outcomes and higher mortality than those unrelated to AF. About 25 percent of strokes have cardio-embolic genesis and AF is the leading cause [22]. This pathophysiological mechanism usually causes embolic occlusions on large-caliber cerebral vessels. Emboli mainly occlude distal arterial branches within the brain, causing superficial infarcts that appear triangular in shape with the base of the triangle on the surface of the brain and the apex facing inward. The presence of embolism is suggested by CT (including perfusion sequences) or MRI by the location and shape of the lesion, the presence of wedge-shaped superficial infarcts in several different vascular territories, hemorrhagic infarction, and visualization of thrombi within the arteries [23]. In this setting, as suggested by the literature, CTP shows the greatest sensitivity in detecting cardio-embolic stroke [24]. Our results extend this diagnostic trend of CTP on PCS as well.

In our study, dyslipidemia recorded particular results (OR = 0.285, *p* = 0.019). Usually, dyslipidemia and more generally atherosclerotic processes amplify and increase endothelial dysfunction and inflammatory pathways at that level. These processes lead to large-vessel strokes from atherosclerotic causes, while the mechanisms of arteriolosclerosis, caused by dyslipidemia and other risk factors, cause strokes with lacunar genesis. The literature data have shown how in lacunar syndromes the CTP has low sensitivity, which is particularly marked at the level of the posterior cranial fossa [25].

Our study showed that the finding of visual field deficit has a strong power of association with the finding of alterations in CTP (OR = 3.372, *p* = 0.046). This result could be linked to the site of alteration, namely the occipital lobe. CTP is known to be magnified in ischemic lesions affecting cortical sites [25]. Our result suggests that this clinical element is a key factor and it guides the clinician in the interpretation of perfusion maps. We also classified the patient neurological deficit according to NIHSS into minor stroke (NIHSS ≤ 5) and moderate/severe stroke (NIHSS > 5), highlighting a relation between major neurological deficit and positive perfusion maps (OR = 4.054, *p* = 0.030). According to the literature, a higher NIHSS score is suggestive of a stroke with a larger caliber vessel occlusion. A strong correlation between the baseline NIHSS score and the ischemic volume estimated by CTP is known, thus it follows that NIHSS is a reliable predictor of perfusion deficits in acute ischemic stroke [18]. Strokes with higher NIHSS usually have a cardio-embolic genesis. As previously stated, the cortical regions represent the most sensitive territory for CTP.

Several limitations are noted in this study: the limited sample size and the retrospective character of this study. The limit of NIHSS scale in case of PCS may lead to an underestimation of the neurological signs of such patients.

## Conclusion

Our study showed that CTP may be a valid asset in detecting acute PCS, showing moderately high sensitivity which was higher than that of NECT alone or CTA. Among the CTP maps, MTT proved to be the most sensitive for identifying PCS. Clinical factors associated with the presence of perfusion alterations on CTP included atrial fibrillation, major neurological deficit (NIHSS > 5), visual field deficit, and the absence of dyslipidemia. These findings contribute to the growing body of evidence supporting the use of perfusion imaging in acute stroke and underscore the potential added value of CTP in cases of posterior strokes.

## Data Availability

The data will only be made available from the corresponding author upon reasonable request.
